# Towards large scale hybrid QM/MM dynamics of complex systems with advanced point dipole polarizable embeddings†
†Electronic supplementary information (ESI) available. See DOI: 10.1039/c9sc01745c


**DOI:** 10.1039/c9sc01745c

**Published:** 2019-06-11

**Authors:** Daniele Loco, Louis Lagardère, Gérardo A. Cisneros, Giovanni Scalmani, Michael Frisch, Filippo Lipparini, Benedetta Mennucci, Jean-Philip Piquemal

**Affiliations:** a Sorbonne Université , CNRS , Laboratoire de Chimie Théorique, LCT , Paris , France . Email: jean-philip.piquemal@sorbonne-universite.fr ; Email: daniele.loco@lct.jussieu.fr; b Sorbonne Université , CNRS , Institut Parisien de Chimie Physique et Théorique, IP2CT , Paris , France; c Sorbonne Université , Institut des Sciences du Calcul et des Données, ISCD , Paris , France; d University of North Texas , Department of Chemistry , TX , USA; e Gaussian, Inc. , Wallingford , CT , USA; f Univerisita di Pisa , Dipartimento di Chimica e ChimicaIndustriale , Pisa , Italy; g Institut Universitaire de France, IUF , Paris , France; h The University of Texas at Austin , Department of Biomedical Engineering , TX , USA

## Abstract

In this work, we present a general route to hybrid Quantum Mechanics/Molecular Mechanics (QM/MM) Molecular Dynamics for complex systems using a polarizable embedding. We extend the capabilities of our hybrid framework, combining the Gaussian and Tinker/Tinker-HP packages in the context of the AMOEBA polarizable force field to treat large (bio)systems where the QM and the MM subsystems are covalently bound, adopting pseudopotentials at the boundaries between the two regions. We discuss in detail the implementation and demonstrate the global energy conservation of our QM/MM Born–Oppenheimer molecular dynamics approach using Density Functional Theory. Finally, the approach is assessed on the electronic absorption properties of a 16 500 atom complex encompassing an organic dye embedded in a DNA matrix in solution, extending the hybrid method to a time-dependent Density Functional Theory approach. The results obtained comparing different partitions between the quantum and the classical subsystems also suggest that large QM portions are not necessary if accurate polarizable force fields are used in a variational formulation of the embedding, properly including the QM/MM mutual polarization.

## Abstract 2

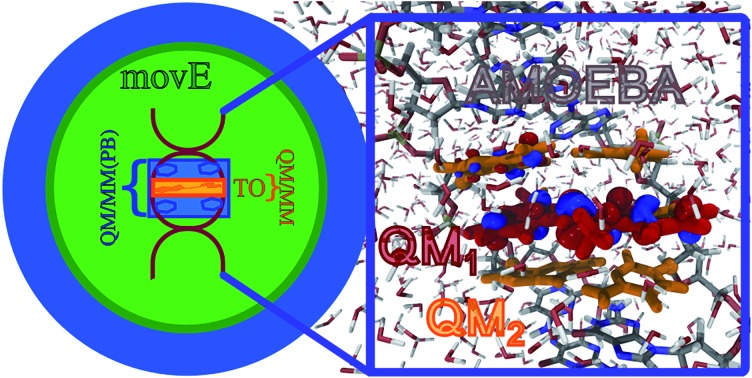
Hybrid DFT(Gaussian)/AMOEBA(Tinker/Tinker-HP) polarizable molecular dynamics including the QM/MM mutual polarization on large complex systems. Example of the thiazole orange dye buried in a DNA double helix, embedded in a sphere of water (16 500 atoms).

## 1 Introduction

Hybrid QM/MM approaches, combining quantum mechanical (QM) and classical atomistic models based on molecular mechanics (MM),^1^–^4^ are nowadays widely applied to the modeling of biological and other complex systems. Such models combine a high resolution, fully atomistic description of a complex system with a focused approach that introduces a distinction between the relevant portion of the system, usually named its *target*, and an *environment* that, while playing a fundamental role in tuning the properties of the target, does not participate directly in the process of interest. This has two main advantages. First, the very high dimensionality of the computational problem is largely reduced and the set of information extracted from the calculations becomes of a similar order of magnitude to, and more pertinent to, its experimental counterpart. Second, it is very cost effective, as while retaining a description that represents the system in its full complexity, the vast majority of the system is described with fairly inexpensive MM, while the computational effort is concentrated on the target, that can be described at an accurate, though expensive, QM level of theory, which would not be applicable to the whole system. Finally, QM/MM methods become mandatory when dealing with extremely complex and large systems involved in processes which require the explicit treatment of the electronic structure problem such as enzymes or photoresponsive proteins.^5^–^9^

The definition of the target of such systems is not always a straightforward task, especially when the target and the environment are linked together by a covalent bond. As a matter of fact, in biological matrices (*e.g.*: proteins, DNA) several covalent bonds between the QM and the classical subsystem can be involved. It is evident that in all these cases, the way the system is partitioned becomes crucial to achieve a correct description of the process of interest. For instance, peculiar strong specific interactions between a small portion of the environment and the target may require to extend the QM treatment to a larger part of the system.^10^–^13^

The modeling of mixed QM/MM bonds has been a long-lasting argument of debate in the community and several different approaches have been developed in the years to address this issue.^3^,^4^,^14^,^15^ All these models have to solve, in some way, three main problems. First, cutting a covalent bond results in a QM atom with an unrealistic, highly reactive, unsaturated valence configuration, which can introduce strong artifacts in the simulation. Such an atom has to be capped in order to complete its valence. Second, the QM density at the boundary can be overpolarized when an electrostatic embedding is applied, which can be aggravated when a polarizable embedding is used as also the classical part can overpolarize. Third, the bonding terms in the classical MM force fields involving atoms from both subsystems have to be carefully selected in order to avoid double-counting of interactions.

Three different classes of boundary schemes can be found in the literature. A first class is made by link-atom schemes, where an additional (usually hydrogen) atom is introduced in the QM subsystem to cap the missing valence. Such an additional atom is not part of the real system and the charge next to the QM subsystem may be blurred or redistributed.^16^–^18^ The second one is made by localized orbital schemes,^1^,^15^,^19^ which employ hybrid orbitals placed at the boundary to cap the QM region, keeping some of them frozen. Finally, the third class uses a capping potential to terminate the QM region.^20^–^23^ In this work we will focus on a specific version of this third class, the *pseudobond* scheme.^20^–^22^

This implementation is integrated in our polarizable QM/MM method, based on the AMOEBA polarizable (and multipolar) force field,^24^–^29^ which interfaces the Gaussian and Tinker packages to achieve molecular dynamics (MD) on the adiabatic ground state energy surface of the QM/MM system. This new implementation is extensively tested on a set of oligopeptides, for which we discuss the energy conservation along hybrid ground state trajectories. Three test systems of increasing complexity are considered to show the stability of the method: an alanine dipeptide, a 6 aminoacid oligopeptide (SAPPAS) and a 20 aminoacid oligopeptide (PEP). We then detail the applicability of our method to the computation of excitation properties on a large solvated system encompassing a chromophore intercalated in DNA for which we will compute electronic excitations within a sequential approach based on the hybrid QM/MM molecular dynamics.

## 2 Polarizable QM/MM with the AMOEBA force field

We will first present the details of the pseudobond scheme adopted in the new implementation designed to treat QM/MM boundary regions in the hybrid QM/AMOEBA self-consistent field (SCF)-based method, implemented in a development version of Gaussian 09 (ref. 30) interfaced with the MM/MD Tinker and Tinker-HP^31^,^32^ suite of programs. A brief recall of the very fundamental equations of the method is then presented. A more complete derivation of the QM/AMOEBA equations can be found in the literature,^33^–^37^ as well as a complete description of the polarizable (and multipolar) AMOEBA FF.^27^–^29^

### 2.1 The pseudobond scheme

When the target and the environment are chemically bonded, the QM/MM treatment of the system requires one to cut at least one covalent σ bond A–B. A and B refer to boundary atoms of the environment and the active part, respectively. In the pseudobond (PB) approach the A_PB_–B bond is formed by replacing the A atom with a one-free-valence boundary atom (A_PB_) to complete the open valences of the bonds that cut across the QM/MM boundary. A_PB_ is then included in the QM subsystem and customized effective core potentials (ECPs)^38^–^42^ are used to treat A_PB_. In the PB approach the ECP is designed to simultaneously reproduce the properties of the bond that is replaced (charge, bond strength/distance, *etc.*) and results in a minimal perturbation on the original system. From now on, the QM–MM covalent bond is called a *pseudobond* (PB) and the atom selected as the boundary atom and included in the (usually closed-shell) QM subsystem is the PB atom.

In this work we apply the recently proposed PBs by Yang and co-workers.^22^,^43^ The use of PBs for QM/AMOEBA hybrid simulations has been previously reported and implemented in the LICHEM code.^44^,^45^ Here, we employ the same approach as developed by Kratz *et al.*^44^

The ECPs are of the form
1

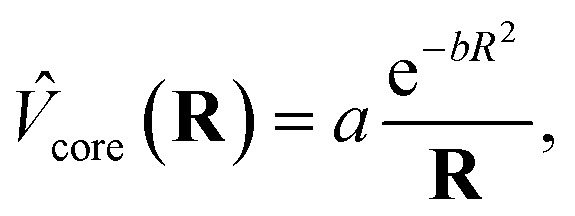

with *a* and *b* being empirical parameters optimized for specific types of pseudobonds, like C_ps_–C, C_ps_–N and C_ps_–C=O, where the subscripts ps indicate the atom bearing the pseudopotential. In this formulation the seven-valence-electron boundary atom has its own basis set, namely the STO-2G basis set with four parameters instead of six since the same exponents are assigned to the s and p basis functions.^21^,^22^

A remarkable advantage of the PB approach is that it does not require any additional term to be implemented in the electronic structure code, as long as such a code is able to handle ECPs, which is usually the case. On the other hand, a significant effort has been put into the design of a flexible QM/MM interface, capable of handling the double quantum and classical nature of the PB atom. Such a duality requires in fact the handling of its associated electronic density within the electronic structure code (Gaussian 09), and the inclusion of bonding interactions involving quantum atoms in the MD code (Tinker), which normally ignores such class of atoms in the energy computation.

### 2.2 Variational energy functional of the QM/AMOEBA embedding

The QM/AMOEBA variational energy functional is defined as^46^
2

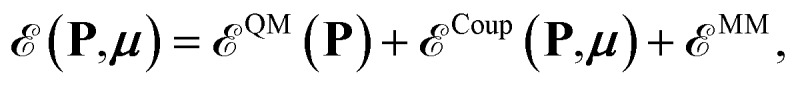

where 
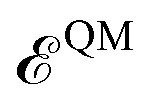
 is the internal energy of the QM subsystem, and 
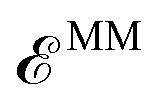
 that of the classical subsystem treated with the AMOEBA force field, while 
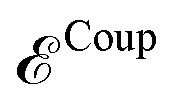
 represents the coupling between the quantum and the classical subsystems. For each energy component in eqn (2), we write explicitly its functional dependence on the variational degrees of freedom that are simultaneously optimized during the SCF procedure, namely the one-body electronic density matrix **P** and the induced dipole moments ***μ*** at the classical polarizable sites.

The QM/MM coupling term is further divided into two different contributions
3

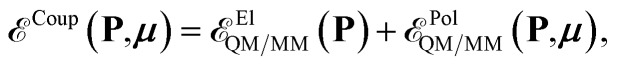

which represent, in order, the electrostatic interaction between the AMOEBA static multipoles (up to quadrupoles) and the QM density and the polarization interaction of the QM density and the AMOEBA induced dipoles^33^,^34^ Here, dispersion/repulsion interactions between the classical and quantum subsystems are included in the 
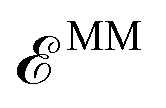
 term, and they are treated using the AMOEBA “softer” buffered 14–7 functional form.^27^

**Fig. 1:**
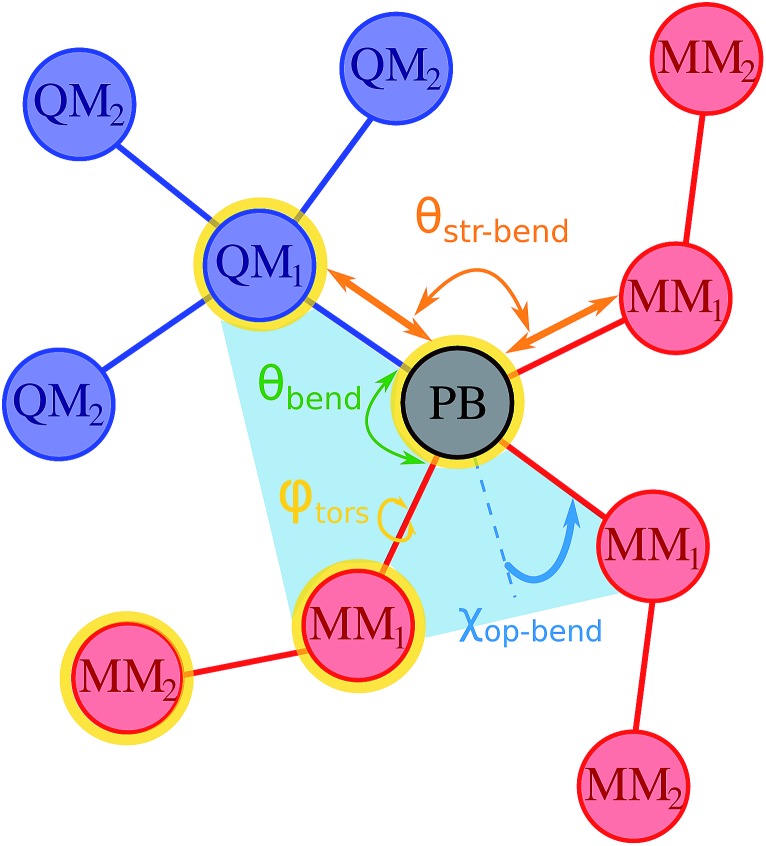
Pictorial representation of bonding terms occurring between the QM (blue circles) and the MM (red circles) subsystems across the pseudobond atom (PB, black circle). Such hybrid contributions are of the type {QM_*Y*_}^*i*^–PB–{MM_*X*_}^*j*^, with the subscripts X and Y ranging from 1 to 2, representing the number of bonds between the QM (MM) and the PB atom. The superscripts *i* and *j* indicate the number of QM and MM atoms involved in the bonding interaction, with 2 ≥ *i* ≥ 0 and 2 ≥ *i*, *j* ≥ 1, depending on the interaction. Such interaction terms are those defined in the classical AMOEBA FF, occurring at least between three atoms, except for the stretching term occurring between the PB atom and the bonded MM_1_ classical sites. Some of all these terms are shown as the angle bending (green), out-of-plane bending (blue), dihedral torsions (yellow), and stretching-bending coupling interactions (orange). Torsion–torsion coupling and π-torsions are also included in the QM/MM Hamiltonian but not represented.

Finally, the last term in eqn (2), 
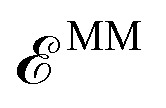
, includes all the electrostatic interactions between the classical sites and all the bonding interactions of the classical FF. The bonding interactions which are accounted for in 
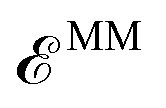
 include also those bonding terms arising from the QM and MM atoms connected across QM/MM boundary regions. To compute these bonding terms between classical and quantum atoms connected through a PB atom, the PB atom itself and the QM atoms involved are treated as classical atoms, using the atomic parameters defined in the classical FF for the atom type required. In Fig. 1 the bonding interactions are depicted in a schematic manner, representing respectively: bond stretching, angle bending decomposed into in-plane and out-of-plane components, bond-angle cross terms, and torsional rotation, including torsion–torsion coupling terms.

The 
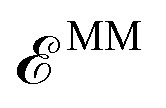
 term does not depend on either the electronic density or the induced dipoles, and for this reason it does not enter the SCF procedure. It is worth noting that the PB atoms do not need any classical multipoles or polarizabilities since the description of the PB atoms is included in the nuclear and electronic part of the QM subsystem and thus any electrostatic and polarization interactions involving the PB atoms are included in the hybrid terms of eqn (3).

Taking advantage of the variational formulation, the coupled QM/AMOEBA equations needed to plug the QM/MM scheme in the quantum chemistry method of choice have been derived, and an *effective* QM/AMOEBA Fock (or Kohn–Sham, depending on whether Hartree–Fock or DFT theory is concerned) matrix can be obtained as the gradient of the energy functional in eqn (2) with respect to the density matrix.^33^

Analytical derivatives of the QM/AMOEBA energy are obtained by differentiating the energy functional of eqn (2) with respect to both classical and quantum nuclear coordinates. The hybrid QM/MM forces can then be used to perform QM/MM Born–Oppenheimer molecular dynamics (BOMD).^34^ An extended Lagrangian approach (XL-BO), in the formulation proposed by Niklasson and co-workers,^47^–^50^ is applied to improve the guess for the electronic density at each integration time step of the dynamics to speed-up the convergence of the SCF procedure.^34^

### 2.3 Excitation properties

The implementation has also been extended to the simulation of electronic excitations, using a time-dependent DFT (TDDFT) scheme.^33^ The whole spectrum of the excitations of interest is determined in a single step calculation, by solving for the poles of the TDDFT response function. An additional contribution in the response equations is introduced by the inclusion of the polarizable embedding operator in the Hamiltonian, which is computed through the transition densities corresponding to the different excitations. Such a term takes into account the dynamic response of the polarizable environment.

The detailed derivation of the TDDFT equations in a polarizable environment can be found elsewhere.^51^ Here we only report the final modified TDDFT equations:^52^
4

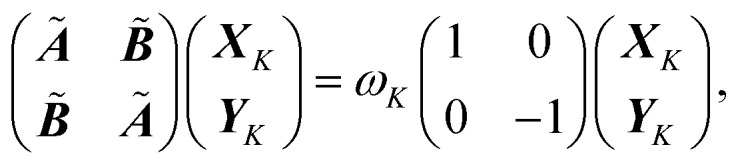

and observe that the matrices ***Ã*** and ***B[combining tilde]*** incorporate the effects of the polarizable embedding in two ways. First, the integrals that they contain are computed using molecular orbitals (MOs) determined in a QM/MM SCF computation. Secondly, ***Ã*** and ***B[combining tilde]*** include an explicit term involving the classical induced dipoles, namely:^33^
5

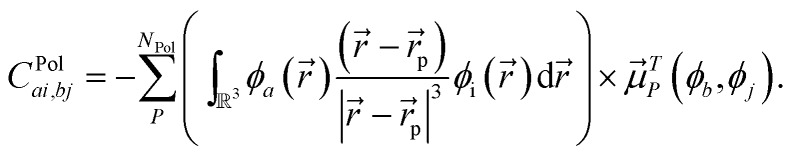

where the *a*, *b* and *i*, *j* indices refer to occupied and virtual MOs, respectively. Such classical induced dipoles are computed at each step of the iterative procedure usually employed to solve the Casida equations.

To account for the further environment effects due to the relaxation of its polarization in response to change in the QM density in the excitation process, a so-called “state-specific” correction,^53^,^54^ completely equivalent to the so-called “corrected Linear Response” (c-LR) scheme originally developed for polarizable continuum models,^55^ was implemented in the QM/AMOEBA method.^33^ Within this framework, a relaxed density matrix is calculated for the excited state of interest and the corresponding excitation energy is corrected for the interaction of the corresponding electric field and the induced dipole moments within the environment.

## 3 Numerical tests

The numerical stability of the pseudobond approach has been tested by analyzing the energy conservation along short trajectories of three oligopeptides: an alanine dipeptide (ALA), an hexapeptide (SAPPAS, amino acidic sequence: Ser–Ala–Pro–Pro–Ala–Ser) and a twenty aminoacid oligopeptide composed of each of the natural amino acids (PEP, amino acidic sequence: Ala–Gly–Val–Leu–Ile–Pro–Ser–Thr–Cys–Phe–Tyr–His–Trp–Asp–Asn–Glu–Gln–Met–Lys–Arg).

The oligopeptides are arbitrarily partitioned in a QM portion, modeled with DFT, and an MM one, modeled with the AMOEBA polarizable potential. The QM portion consists of one of the alanine residues for ALA, two central prolines for SAPPAS and a four aminoacid sequence (tyrosine–histidine–tryptophan–aspartate) for PEP. The boundaries between the QM and the classical portions in the oligopeptide are treated with the adequate PB, depending on whether the PB formed on the QM side is a C_PB_–N or C_PB_–C=O bond, where C_PB_ indicates the carbon atom bearing the pseudopotential, and the parameterized basis set, and included in the QM portion as the boundary atom. Simulations where a QM/MM partition is performed within the PB scheme will be referred to in the rest of the manuscript with the tag QM/MM(PB).

When the PB approach is applied, the fixed multipoles and the atomic polarizability of the classical atoms directly bonded to the PB atom (MM_1_ sites according to Fig. 1) are set to zero and the point charges are distributed on the MM_2_ atoms to balance the total charge of the system, as suggested by Zhang and co-workers.^20^,^21^

ALA is the simpler and smaller system, and for this reason it is employed to fully explore the capabilities and the limits of the PB approach. For such a purpose two sets of simulations are performed on ALA: (i) gas-phase dynamics and (ii) dynamics in a small droplet of water, treated with the AMOEBA potential. For each set of simulations the QM/MM(PB) dynamics is compared with a full MM trajectory, where the ALA is described completely with the AMOEBA potential, and a full QM one, where the ALA is full DFT. The total energy conservation is compared between the different models used to describe the ALA system and also their effects on some structural parameters, whose adequate description could be crucial in the modeling of a more realistic chemical problem.

Energy conservation is discussed for the QM/MM(PB) dynamics of SAPPAS and PEP only in a water droplet (AMOEBA water), and compared with results obtained from a trajectory performed with a full MM oligopeptide. Those two systems, even though small compared to real-life MD applications, are indeed more complex than the simple ALA dipeptide. For this reason, and because the AMOEBA FF is parameterized only for simulations in solution, gas-phase tests on SAPPAS and PEP are of little interest to draw significant conclusions on the physics of the PB scheme.

All the trajectories are run for 2 ps at the B3LYP/6-31G(/AMOEBA) level of theory, in the NVE ensemble, with an integration time-step δ*t* = 0.5 fs and initial velocities sampled from a Maxwell–Boltzmann distribution at 80 K. The integrator applied is the velocity Verlet. The low temperature is chosen to minimize the noise in the energy fluctuations.^34^

### 3.1 Alanine dipeptide

In QM/MM(PB) simulations of the ALA system the QM/MM partition occurs through the α-carbon atom of the amide bond between the two alanine residues, which is then defined as the PB atom. The pseudobond formed in this way is a carbon–carbon σ-bond between the PB atom and the neighboring sp^2^ carbon of the carbonyl group in the QM portion.

**Fig. 2:**
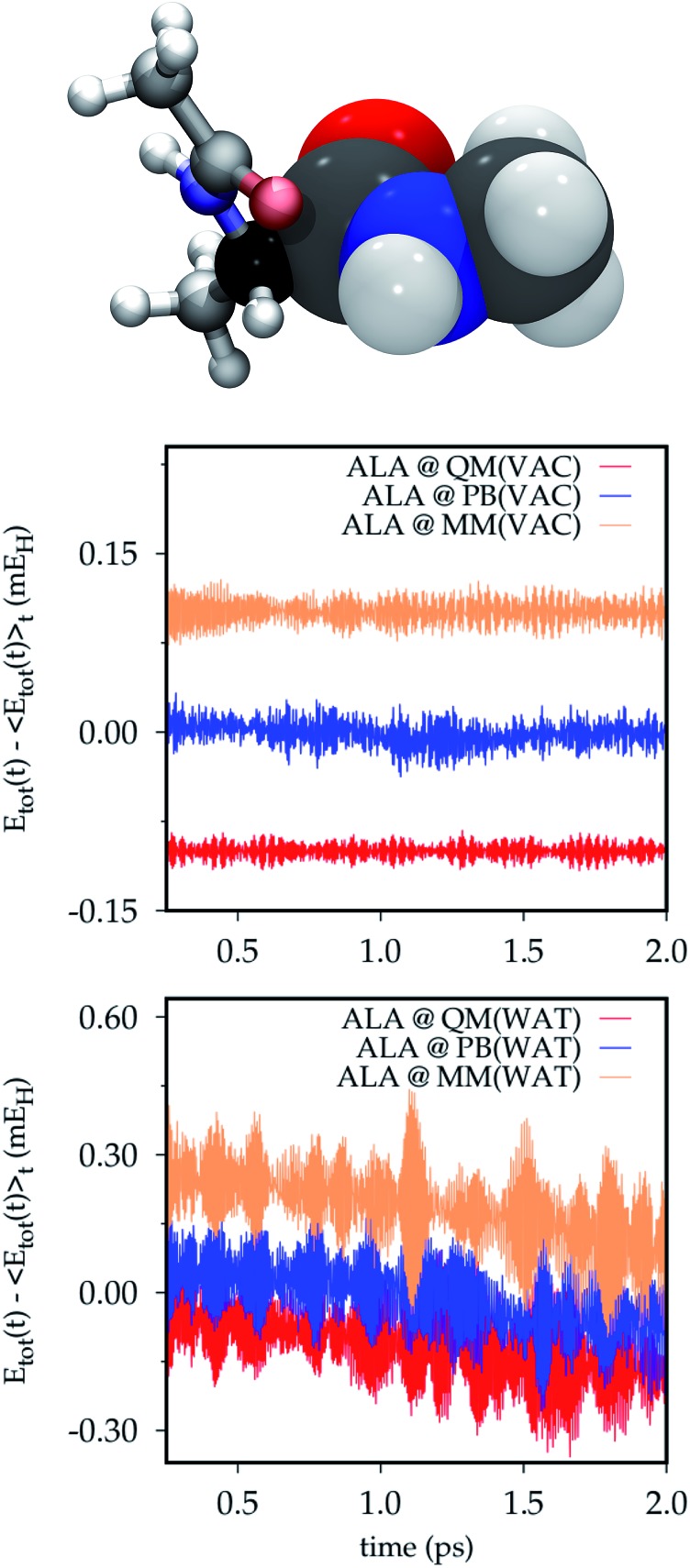
(Top) Representation of the ALA system. The PB atom is represented in black, and the MM portion of the peptide is represented as ball-and-sticks, while the quantum part is represented as van der Waals spheres. (Bottom) Total energy conservation along the different 2 ps NVE trajectories where: (i) the ALA dynamics is performed *in vacuo*, treating the dipeptide as full QM (@ QM(VAC)), with the PB approach (@ PB(VAC)) and as full MM (@ MM(VAC)); (ii) the ALA dynamics is performed in a droplet of water (@ QM(WAT), @ PB(WAT), @ MM(WAT)). The water solvent and the classical portions of the amino acids are treated with the AMOEBA potential, while the QM systems are treated at the B3LYP/6-31G level of theory. The total energy is reported in milli-hartree (mE_H_) and shifted by the average value computed along the trajectory () and shifted by the average value computed along the trajectory (〈*E*_tot_(*t*))〉_*t*_), while the time on the *x* axis is reported in pico-seconds (ps). ALA @ QM and ALA @ MM have been shifted by –0.1 mE_H_ and +0.1 mE_H_ respectively, to avoid the superposition of the different time series.

In Fig. 2 the partitioned system is depicted and the total energy *E*_tot_ time-series of full QM, QM/MM(PB) and full MM trajectories is reported. Either *in vacuo* (@ QM(VAC), @ PB(VAC) and @ MM(VAC)) and in the small droplet of classical AMOEBA water (@ QM(WAT), @ PB(WAT) and @ MM(WAT)) the drift in the total energy is barely noticeable, even in the very small scale of the energy fluctuations due to the low temperature (∼±10^–5^–10^–4^ hartree (E_H_)). Both the drifts and the oscillations in the total energy appear slightly larger in the simulation in solution. This is due to the increased complexity in the dynamics, which stems from the presence of the solvent. Overall, the drift is still remarkably small (∼10^–4^ E_H_). Furthermore, no significant differences can be observed in the behaviors of the full QM, QM/MM(PB) and full MM simulations, meaning that the introduction of the PB scheme does not affect the total energy conservation significantly.

The “*in vacuo*” trajectories are analyzed more in detail from a structural point of view. To have a larger sampling the dynamics have been extended to 4 ps. The energy conservation of the elongated dynamics is still consistent with what was observed for the shorter ones and the equivalent analysis is reported in the ESI (Section S1†).

The three compared trajectories all start from the same geometry but exhibit different initial velocities, and thus they can, in principle, evolve in different regions of the PES. Since they evolve for a short time, and because of the relatively “simple shape” of the PES for the ALA system and the low temperature of the simulation, this effect is not expected to play a major role in the structural differences that can be detected.

**Fig. 3:**
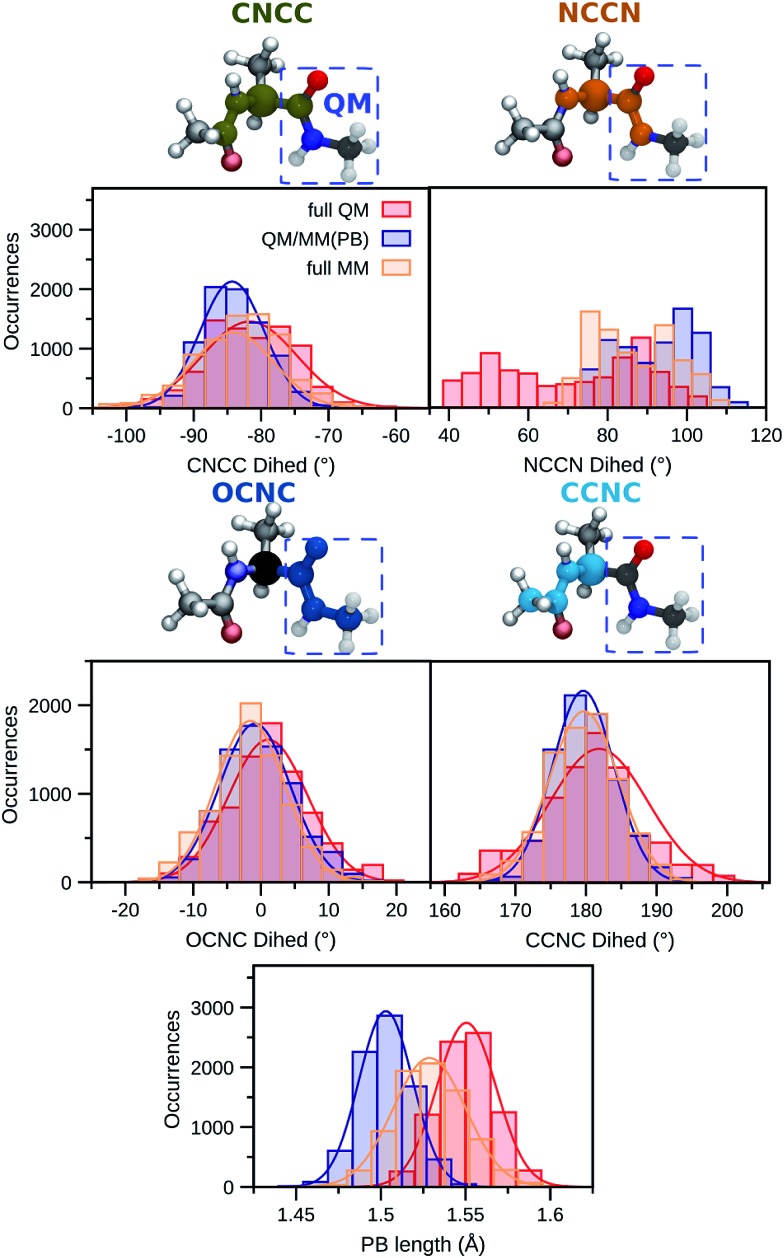
Comparison between selected structural features of ALA along a full MM (yellow), full QM (red), and the hybrid QM/MM(PB) (blue) dynamics. The distributions are drawn from 8000 snapshots extracted from 4 ps trajectories of ALA in the gas-phase. The structural parameters analyzed are: two hybrid QM/MM dihedrals occurring across the PB defined in the QM/MM partition (see Fig. 2), named CNCC (green) and NCCN (orange), the OCNC (blue) dihedral, which is fully QM in the QM/MM(PB) dynamics and the CCNC (cyan) one, which involves the PB atom and it is fully MM in the QM/MM(PB) dynamics. The length of the PB formed in the QM/MM partition scheme is also analyzed. The continuous lines are Gaussian fitting to the distribution.

First, two dihedral angles are analyzed as they are involved in bonding terms between quantum and classical atoms connected through the PB atom within the QM/MM(PB) dynamics. These dihedrals are reported in Fig. 3 and named CNCC (green) and NCCN (orange). We also report their distributions along the full QM (red), QM/MM(PB) (blue) and full MM (yellow) dynamics in the gas-phase. For the CNCC dihedral, where two of the involved atoms are in the MM region, the full MM and QM/MM(PB) distributions are almost overlapping.

The scenario is quite different for the NCCN dihedral, where the two involved atoms are in the QM region (see Fig. 3). In this case the full QM distribution shows two main domains, centered around 50 and 90°, respectively, while both the QM/MM(PB) and the full MM span a smaller domain of dihedral angles, between 70 and 110°. The QM/MM(PB) and full MM distributions are mostly overlapping, even though the intensities of the single bins can vary a lot between them, and they also exhibit a similar shape, with two separate peaks in both cases centered around the intervals 70–80° and 90–100°.

The large difference between the full QM results and the other distributions can be certainly attributed to the mismatch between the DFT and the AMOEBA potentials, AMOEBA being fitted on a higher level of QM accuracy.^27^ The comparison also shows that the overall quality of the QM/MM dynamics is not significantly deteriorated with respect to the one based on the AMOEBA FF by the PB approach. To conclude the analysis of dihedrals in the QM/MM(PB) trajectory, two further ones, namely, one involving only QM atoms (OCNC, blue) and one only involving MM atoms and the pseudoatom (CCNC, cyan) are analyzed. In both cases the comparison between the distributions of such angles in the three different trajectories shows a good agreement, especially for the OCNC dihedral, where the QM/MM(PB) distribution strongly resembles a hybrid between the full MM and the full QM one. The OCNC case, however, points out that the effect of the PB, even though reasonably small, is not completely negligible in the close proximity of the QM/MM boundary.

A similar analysis has been carried out on the length of the bond which corresponds to the PB length defined in the QM/MM(PB) simulation, compared to the length of the corresponding bond in the full QM and full MM dynamics. The resulting distributions (Fig. 3) show a Gaussian behavior, with the full QM case (red) having a C–C bond which is on average longer than that of the corresponding PB (in blue), with an average difference of ∼0.05 Å. The full MM value (yellow) lies in between the QM/MM(PB) and the full QM. Considering the full QM simulation as a reference, the PB approach is reasonably in accordance. The differences in the bond lengths are well motivated by the effect of the smaller basis set for the valence electrons and the pseudopotential used to treat the core electrons.

It is worth reminding here that these analyses on the QM/MM boundaries represent a limiting case for the method since, as usual in QM/MM, the QM region of interest, where structural parameters can play a fundamental role, is always kept as far as possible from the QM/MM boundary region, in order to minimize the effects of the cut.

### 3.2 Two larger cases: SAPPAS and PEP oligopeptides

The energy conservation within the PB approach is tested also on the SAPPAS and PEP systems on trajectories performed for such systems embedded in small water droplets.

**Fig. 4:**
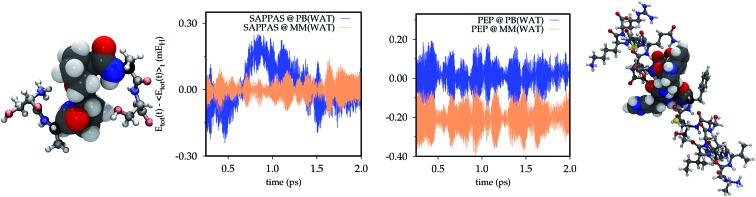
Total energy conservation along the 2 ps NVE trajectories in a small droplet of AMOEBA water where: (i) the oligopeptides are partitioned in a QM/MM scheme with the PB approach (@ PB(WAT)), (ii) the oligopeptides are treated at the full MM level with the AMOEBA FF (@ MM(WAT)). The QM systems are treated at the B3LYP/6-31G level of theory, coherently with the ALA tests. The total energy is reported in millihartree (mE_H_) and shifted by the average value computed along the trajectory () and shifted by the average value computed along the trajectory (〈*E*_tot_(*t*))〉_*t*_), while the time on the *x* axis is reported in pico-seconds (ps). For PEP the full MM simulation has been shifted of –0.2 mE_H_ to avoid the superposition of the different time series. On the left and right side a representation of the SAPPAS and PEP systems is reported, respectively.

For the SAPPAS hexapeptide two PB atoms are defined, one on each of the two alanine units (see Fig. 4, top panel). In one case the PB replaces the α-C of a C–N bond in the quantum part, while in the other a C–C=O bond is replaced, as for ALA. In Fig. 4 (Bottom panel) the SAPPAS total energy conservation along the full MM trajectory (yellow) is compared with that of the QM/MM(PB) dynamics (blue). The QM/MM(PB) energy time-series (SAPPAS @ PB(WAT)) slightly deviates from the full MM starting a positive drift after 0.5 ps of simulation. It suddenly disappeared with an opposite sign drift. Around 2 ps the drift has been removed. A longer 6 ps trajectory is analyzed in the ESI (Section S2†), showing a negative drift reappearing after 3 ps. The drift appears linear in time and of very small entity, with a negligible largest energy deviation from the average of ∼0.3 mE_H_ (less than 0.2 kcal mol^–1^).

Finally, as a last numerical test, the PEP oligopeptide (see Fig. 4, top panel) trajectory is analyzed. The QM/MM(PB) total energy conservation (blue) is reported in comparison with that of the full MM dynamics (yellow) shifted by –0.2 mE_H_ to avoid the superposition of the two time series. In this case, as in the ALA dynamics, no significant effect on the total energy conservation can be seen when the PB approach is applied. For PEP the fluctuation in the total energy is generally larger than those of ALA and SAPPAS, due to the larger dimension of the system (WAT simulations involve water boxes of: 630 molecules for ALA, 473 molecules for SAPPAS and 1053 molecules for PEP). Also for PEP a comparison between longer simulations (7.5 ps) is reported in the ESI (Section S2†).

## 4 Thiazole orange absorption when intercalated in DNA

**Fig. 5:**
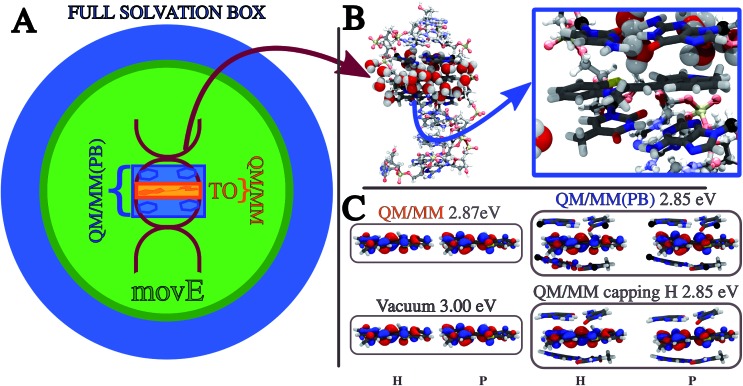
(A) Pictorial representation of the TO dye buried in the DNA double helix, embedded in a sphere of water (16 500 atoms). The different colors represent the differences peculiar to each of the QM/MM partition schemes compared, highlighting the portions of the system included in the QM subsystem of the QM/MM (orange) and the QM/MM(PB) (blue) partition schemes, and the environment selection applied in the *mov*E simulations which differs from that of the other schemes. See the text and Table 1 for a detailed definition of the QM/MM, QM/MM(PB) and *mov*E schemes. (B) The DNA structure is highlighted in a ball-and-stick representation, leaving the first water solvation shell around the TO dye. The QM subsystem defined in this work, tagged as QM/MM(PB) and made up of the TO dye and the four closest nucleobases (NBs) is zoomed out and represented in licorice style in the blue square. The atoms bearing the pseudopotential on the QM/MM boundaries are represented in black. (C) NTOs and excitation energies relative to the ππ* bright excitation of the TO dye embedded in different environments, from vacuum to the QM/MM(PB) partition scheme. Also the results with a different approach to treat the QM/MM boundaries, namely the link atom (QM/MM capping H), are reported. All the NTOs and energies are computed on the initial configuration used for the MD_2_ simulation (see Table 2 and in the text). H and P mean the hole and particle, respectively.

To further test the applicability and reliability of our method, we have investigated the electronic excitations of the Thiazole Orange dye (TO)^56^,^57^ intercalated in a short double helix of DNA solvated in water (see Fig. 5).

In a previous study by some of the authors^58^ the vibronic structure of the absorption band of TO in the biological matrix was simulated applying a sequential scheme based on the QM/AMOEBA MD performed with the Gaussian 09/Tinker interface. The TDDFT including explicit effects of the polarizable environment on the excitation properties was employed. In that study, the system was partitioned in such a way that only the TO dye was treated at the QM level, while the rest of the environment was represented with the AMOEBA FF. In the trajectories used to compute excitation energies, only the TO was allowed to move in a fully classical and frozen environment.^58^

Neither the role of the QM/MM partition scheme employed nor the effects of the frozen environment on the excitation properties have been investigated.

Aiming at shedding more light on these aspects, a new set of simulations is compared to the previous results, applying the PB scheme to assess how the excitation properties are affected by the definition of a larger QM subsystem which goes beyond the TO dye, including also the two pairs of nucleobases (NBs) nearest to the dye. This modified QM/MM partition scheme entangles together the effects on the dynamics of the system and on the resulting excitation properties. The PB approach is applied to the QM/MM boundaries, which are placed between the nucleobase and the sugar connecting the base to the DNA backbone (see Fig. 5). We refer to the new definition of the QM subsystem including the NBs as QM/MM(PB), while the previous one only including TO as QM/MM.

In order to better investigate how the environment relaxation affects the overall results, we performed a third type of MD simulation. Now, all the systems, not only the QM subsystem, is allowed to move; we label the test performed under these conditions as *mov*E.

In Table 1 the notation used to define the different QM/MM partition schemes is reported, together with the composition of the QM and MM subsystems and whether the MM one is frozen or it is moving during the QM/MM MD simulation. In Fig. 5 a pictorial representation of the whole system and of the different schemes applied in this work is reported.

**Table 1:** Notation used in this work to define the different QM–MM partition schemes used for the QM/MM MD: the first column of the table reports the name used to define the partition scheme. In the QM and MM columns the components of the QM and MM partition schemes are reported, respectively. The last column specifies whether the QM and MM portions are free to evolve during the dynamics or are frozen

Simulation tag		QM/MM partition	Dynamics
QM/MM	QM	TO dye	Moving
	MM	DNA + water	Fixed
QM/MM(PB)	QM	TO dye + four NBs	Moving
	MM	Rest of the DNA + water	Fixed
*mov*E	QM	TO dye + four NBs	Moving
	MM	Rest of the DNA + water	Moving

**Fig. 6:**
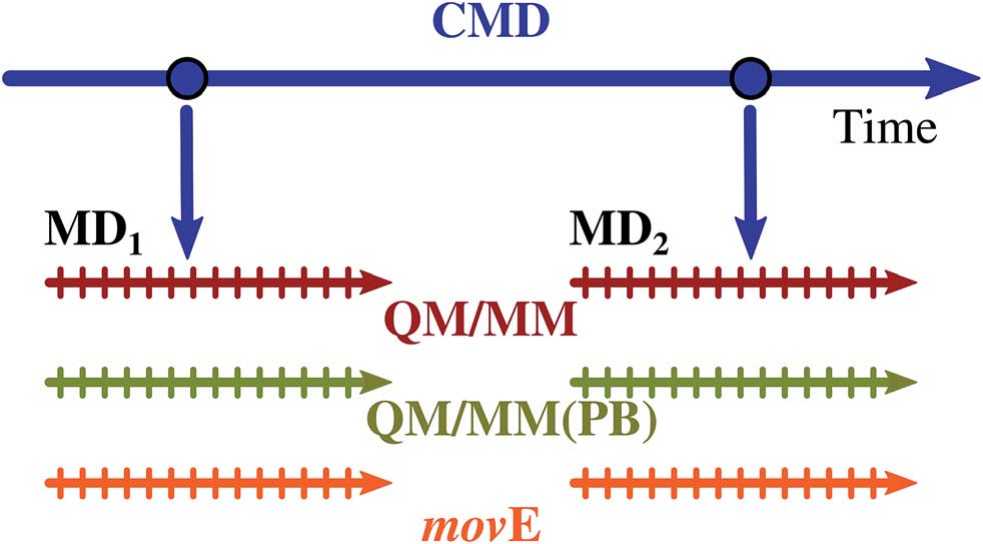
Schematic representation of the procedure followed to compute the MD_1_ and MD_2_ trajectories. Two configurations of the TO dye embedded in the DNA in water solution have been extracted from the classical molecular dynamics (CMD, blue arrow) carried out in ref. 58. From these two configurations the MD_1_ and MD_2_ 2 ps long trajectories have been run within the three different QM/MM partition schemes reported in Table 1: QM/MM, QM/MM(PB) and *mov*E. Each trajectory, for a total of six simulations, has been sampled every 80 fs, removing the first 480 fs as an equilibration time, to extract system-environment configurations to perform TDDFT calculations.

The structures coming from the QM/MM, QM/MM(PB) and *mov*E dynamics are finally used to compute the excitation energies using the same QM–MM partition adopted for the dynamics. Since the present work does not aim at computing the whole absorption band shape of the embedded TO dye, instead of performing several hybrid QM/MM trajectories, and sampling the initial system configuration from a classical dynamics, a reduced set of starting geometries is extracted from the same classical trajectory used in ref. 58, obtaining two starting configurations, far in time from each other as schematically depicted in Fig. 6. The trajectories produced from the two initial configurations selected are named MD_1_ and MD_2_.

The B3LYP/6-31G/AMOEBA level of theory has been used for all molecular dynamics simulations. 2 ps runs in the NVE ensemble with a time step δ*t* = 0.5 fs have been performed to keep consistency with the simulation proposed in ref. 58. No periodic boundary conditions were applied (*i.e.* water droplet), and the initial atomic velocities are assigned through the sampling of a Maxwell–Boltzmann distribution at 300 K.

First the effect of a larger QM subsystem is discussed. In these simulations the classical environment is kept frozen to be consistent with the previous study. The environment, composed of the DNA helix, counterions and water molecules is made up of about 16 500 atoms, most of which are due to the large solvation box (see Fig. 5). In ref. 58 the B3LYP functional has been employed also for the TDDFT calculations which follow the dynamics. When the new QM/MM(PB) partition is applied, the use of the B3LYP functional results in the appearance of charge-transfer (CT) intruder states, delocalizing the TO ππ* transition of interest on the QM NBs. This effect has been investigated computing TDB3LYP/6-31+G(d)/AMOEBA excitations on the snapshots extracted from MD_1_ every 80 fs (see Fig. 6) and the results are reported in the ESI (Section S3†). Excitations computed on the same MD_1_ snapshots but restricting the QM subsystem at the TO dye only, where the CTs cannot take place in the TDDFT calculation, have been used for comparison and the nature of the excitation has been analyzed in terms of the corresponding Natural Transition Orbitals (NTO).^59^

In Section S4 of the ESI† we report the comparison between the B3LYP, CAM-B3LYP, M062X, PBE0, *ω*B97X and *ω*B97XD functionals, showing that the emergence of the intruder CT-like states can be cured by choosing a range-separated hybrid functional.^60^ For this reason the CAM-B3LYP exchange-correlation functional has been selected to compute excitation properties.

The results are reported in Table 2 in terms of the average excitation energies and the corresponding transition dipoles from MD_1_ and MD_2_ run both in the previous QM/MM and the new QM/MM(PB) schemes. The relative differences between the averaged values are also reported. It can be noticed that the differences between the excitation properties computed on MD_1_ and MD_2_ are much smaller in the QM/MM(PB) scheme than those of the QM/MM one. In particular, the average excitation energies in the QM/MM(PB) trajectories differ by only 0.04 eV. The same simulations have been carried out on a third TO-environment configuration extracted from the long classical MD run, giving results very close to MD_2_ both for the QM/MM and QM/MM(PB) schemes. Excitation energies and transition dipole moments have been reported in the ESI (Section S5†).

**Table 2:** Average values of the properties computed along the MD_1_ and MD_2_ trajectories in the different QM/MM schemes are presented. Excitation energies (eV) and electronic transition dipole moments (atomic units) are computed at the TDCAM-B3LYP/6-31+G(d)/AMOEBA level. Each trajectory is sampled every 80 fs (see Fig. 6) to extract a set of system-environment configurations employed to compute the vertical excitations. The average BLA value, evaluated on the snapshot sampled to compute the excitation energy, is reported in pm, together with the correlation coefficient *C*_corr_ computed between the excitation energies and the measured BLAs

	QM/MM				QM/MM(PB)				*mov*E			
	〈*E*_exc_〉	|*μ*|^2^	〈BLA〉BLA〈BLA〉	*C*_corr_	〈*E*_exc_〉	|*μ*|^2^	〈BLA〉BLA〈BLA〉	*C*_corr_	〈*E*_exc_〉	|*μ*|^2^	〈BLA〉BLA〈BLA〉	*C*_corr_
MD_1_	3.02	13.1	–2.5	–0.61	2.91	9.9	–2.7	–0.54	2.82	10.8	0.3	–0.32
MD_2_	2.83	14.3	–0.5	–0.59	2.87	10.7	–0.8	–0.23	2.85	11.1	0.2	0.11
MD_1_–MD_2_	0.19	–1.2	–2.0		0.04	0.8	–2.1		–0.03	–0.03	0.1	

This analysis, based on the two sets of trajectories MD_1_ and MD_2_ and fostered by the third set of simulations reported in Section S5 of the ESI,† indicates that, as expected, allowing the local environment due to the closest NBs to move together with TO reduces the differences introduced by the different initial configurations of the environment.

If we now want to estimate the contribution of the QM–MM partition, separating it from the structural effect related to the NB geometry relaxation, a different comparison has to be done. To do that, we selected a single TO-environment configuration (namely one of the two initial configurations extracted from the classical MD trajectory), and we use it in combination with different embeddings: (i) no environment, defined as vacuum, (ii) the QM–MM partition scheme, where only TO is QM, (iii) the QM/MM(PB) scheme, where also the four closest NBs are treated at the QM level and (iv) substituting the PB approach with link atoms, namely the *QM/MM capping H*. As usually done when the link atom is used, the electrostatic parameters of the classical MM atom that has been substituted by the hydrogen atom have been distributed between the classical atoms connected to it. The hydrogen link-atom has been compared here with the PB scheme only for single point calculations, to provide a wider range of methodologies to the reader, also considering the large diffusion of the link-atom approach.^3^,^4^

The excitation energy, together with the NTOs of the corresponding states, has been reported in Fig. 5. Adding an environment on the selected structure has generally a non-negligible effect on the excitation properties. The 3 eV excitation energy found for the vacuum case is red shifted by roughly the same amount (0.15 eV) in the three different embeddings. As a result, we can observe how the QM–MM partition itself does not significantly affect the excitation properties of TO. This observation is confirmed by the comparisons of the effects of different environments, *i.e.* for instance the (ii) and (iii) embeddings, on the many structures extracted from the MD_1_ (see Section S7 of ESI†). Additionally, the same analysis has been performed modeling the classical environment with the AMBER99sb^61^ force field for DNA and the TIP3P model for water,^62^ finding that within a classical, non-polarizable description of the environment, the computed properties are more sensitive to the definition of the QM and MM subsystems (see Section S8 of ESI†).

A third set of simulations has been performed, where also the environment has been allowed to move together with the QM subsystem (*moving environment* scheme, *mov*E). In this test, a selection of the water solvent has also been performed: two selection radii are tested, first including all the solvent molecules (and the counterions) within a radius of 15 Å from each TO atom, and a second selection with a radius of 23 Å. All classical atoms (roughly 4200 for the small radius and 8400 for the larger one) are moving and treated as polarizable with the AMOEBA potential. As no significant differences in the computed excitation energies have been found between the two selection radii (<10^–2^ eV), only the results on the larger radius simulations are shown in Table 2, while the comparison between the two tested radii is reported in the ESI (Section S6†). As observed for the QM/MM(PB) simulations, also in this case the relaxation of the environment has a larger effect on MD_1_, with a final red shift in the averaged excitation energy of 0.09 eV if the QM/MM(PB) value is used for comparison, and 0.2 eV with respect to that in the QM/MM scheme.

**Fig. 7:**
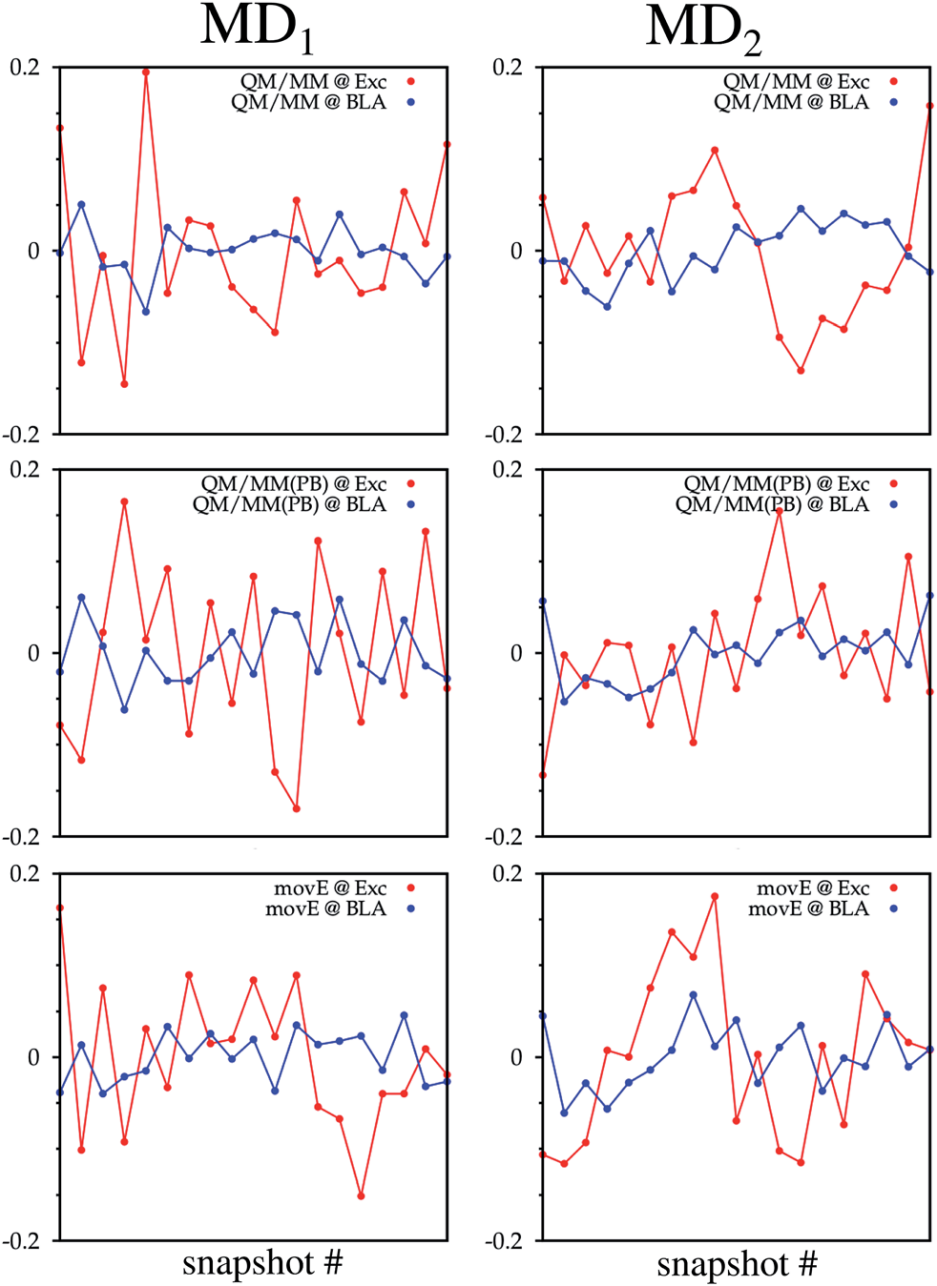
TO ππ* excitation energies (red lines) and BLA (blue lines) fluctuations along the QM/MM, QM/MM(PB) and *mov*E simulation schemes adopted for the MD_1_ and MD_2_ trajectories. On the *x*-axis is reported the snapshot number while on the *y*-axis is reported the difference between the considered properties at the actual snapshot and the average value of the property (either the excitation energy or BLA) along the trajectory. All average values are reported in Table 2.

**Fig. 8:**
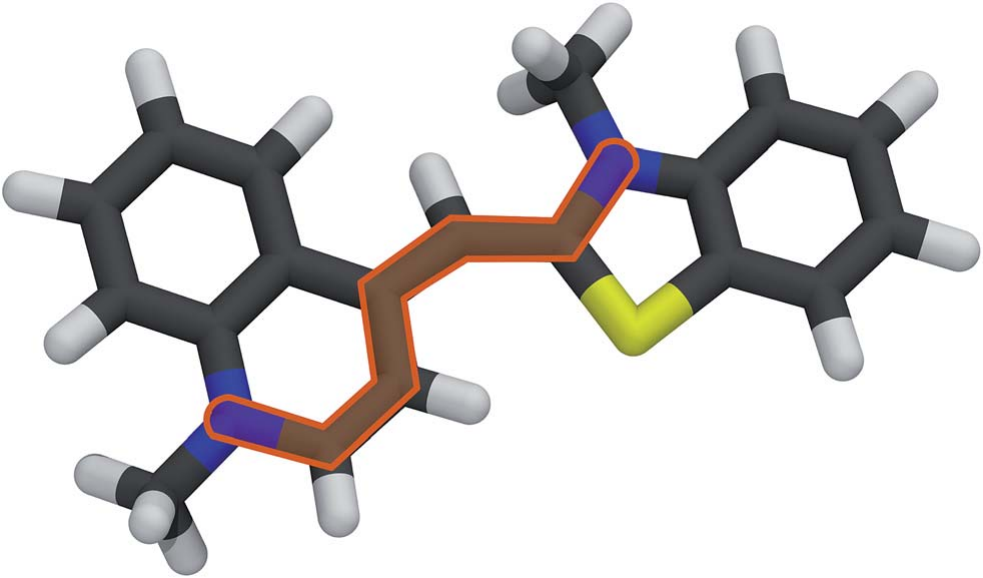
Molecular structure of the TO dye, represented here in licorice style, with the atoms involved in the BLA definition highlighted in orange.

As a key parameter to describe the relation between the internal structure of the TO dye and its excitation properties, the bond length alternation (BLA) defined in ref. 58, and highlighted in Fig. 8 with the TO structure, has also been computed on the structures obtained from all six hybrid trajectories. Their average values are reported in Table 2. Also the correlation coefficients computed between the BLAs and the excitations computed on the sampled snapshots are reported. Negative correlation coefficients highlight an anticorrelation between the two quantities, which can also be observed from the time series reported in Fig. 7. Particularly for the QM/MM simulations a non-negligible anticorrelation can be observed, with a coefficient larger in absolute value than 0.5. For MD_2_ the correlation coefficient becomes significantly smaller also in the QM/MM(PB) scheme. The MD_1_ and MD_2_ BLA values start to be very close to each other only in the *mov*E. In this case, in fact, the constraints due to the fixed environment, together with the differences in the system configurations related to the initial structures are reduced.

## 5 Conclusions

In this work we demonstrated the ability of our hybrid QM/MM MD framework, coupling Gaussian and Tinker/Tinker-HP, to perform QM/MM Born–Oppenheimer molecular dynamics simulations on large (bio)molecular systems including a mutual polarization between the QM and MM regions and treating QM sites that are covalently bonded to the classical subsystem through a pseudobond approach. The main computational aspects of this approach, both concerning its implementation and its application to ground state, DFT/AMOEBA MD simulations, have been discussed together with a detailed analysis of the impact of the boundary regions.

Structural effects have been studied on the QM/MM dynamics of oligopeptides, partitioning the aminoacid sequences into QM and MM portions, linked by covalent bonds. The effects of different QM–MM partitioning schemes have been investigated on the dynamics and the resulting electronic excitation of a dye embedded in a DNA fragment. The comparison shows that an extended QM subsystem going beyond the dye has small effects on the excitation properties if the structure of the whole system is kept the same. This indicates that very large QM subsystems are not required if accurate polarizable force fields such as AMOEBA are used. We also show that a non-polarizable description of the environment using a classical force field may lead to computed properties which are more sensitive to the definition of the QM and MM subsystems. Overall, the importance of a proper handling of the coupled dynamics of the QM subsystem and the environment is also highlighted. If a discussion on the computational cost of such treatment has not been explicitly included in this work, we can add, as a final remark, that (i) Molecular Dynamics with polarizable force fields using distributed multipoles such as AMOEBA can be achieved efficiently on very large systems using modern algorithmics;^32^,^63^,^64^ (ii) the proper treatment of the polarizable environment represents only a small overhead of a few percent on the already expensive SCF procedure of the QM subsystem that dominates the total cost of the simulation.

These results demonstrate that large scale hybrid QM/MM molecular dynamics simulations of complex systems using advanced point dipole polarizable embeddings are now possible offering enhanced accuracy and new perspectives of applications in various fields. Finally, this work paves the way for a production implementation of a polarizable QM/MM scheme to appear in the distribution versions of Gaussian and Tinker/Tinker-HP, for which a more detailed technical description will be given in order to detail the general performance of the model.

## Conflicts of interest

There are no conflicts to declare.

## Supplementary Material

Supplementary information
