# Supplementary material for: Towards large scale hybrid QM/MM dynamics of complex systems with advanced point dipole polarizable embeddings
Source: Chem Sci. 2019 Jun 11;10(30):7200–11. doi: 10.1039/c9sc01745c (PMC6677116; doi:10.1039/c9sc01745c)
Supplement: Supplementary file 1 [file SC-010-C9SC01745C-s001.pdf]

Electronic Supplementary Material (ESI) for Chemical Science.
This journal is © The Royal Society of Chemistry 2019

Towards Large Scale Hybrid QM/MM Dynamics of Complex Systems with Advanced Point Dipole Polarizable Embedding

Daniele Loco,[*,†] Louis Lagardère,[‡,⊥] Gérardo A. Cisneros,[¶] Giovanni Scalmani,[§] Michael Frisch,[§] Filippo Lipparini,[∥] Benedetta Menucci,[∥] and Jean-Philip Piquemal[*,†]

†*Sorbonne Université, CNRS, Laboratoire de Chimie Théorique, LCT, Paris, France.*

‡*Institut des Sciences du Calcul et des Données, ISCD, Paris, France.*

¶*University of North Texas, Department of Chemistry, TX, USA.*

§*Gaussian, Inc., Wallingford, CT, USA.*

∥*Univerisita di Pisa, Dipartimento di Chimica e ChimicaIndustriale, Pisa, Italy*

⊥*Sorbonne Université, CNRS, Institut Parisien de Chimie Physique et Théorique, IP2CT, Paris, France.*

E-mail: daniele.loco@lct.jussieu.fr; jean-philip.piquemal@sorbonne-universite.fr

S1 Extended Dynamics of the ALA test system

We report in Fig. S1 the total energy conservation analysis on the extended 4 ps trajectories of the ALA dipeptide in gasp-phase. The analysis is analogous to those performed in the main text on all the test system.

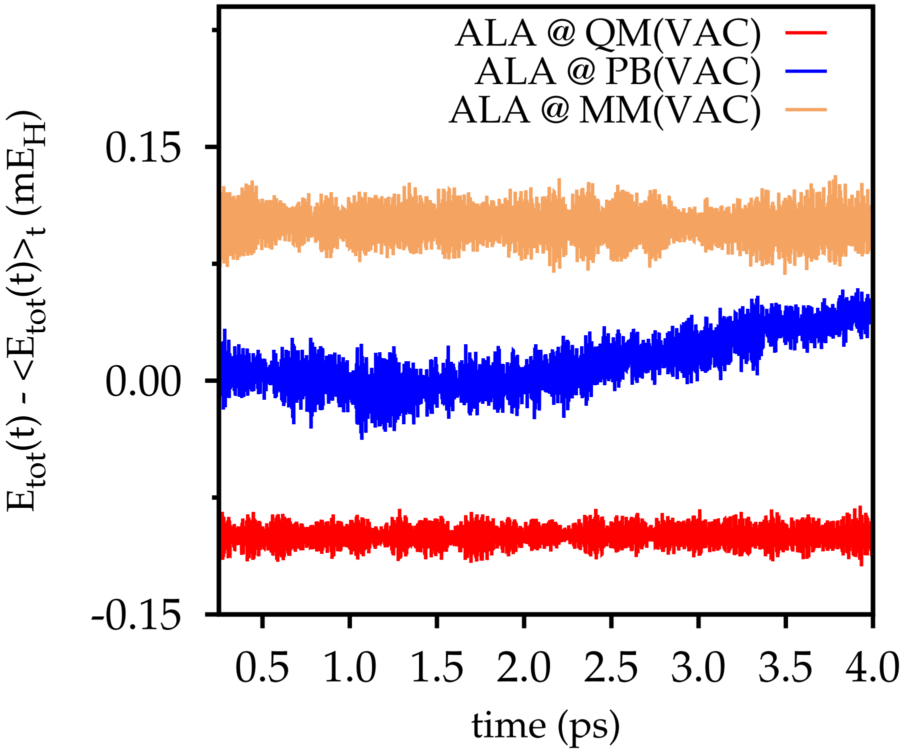

Figure S1: Total energy conservation along the different 4 ps NVE trajectories where: i) the ALA dynamics is performed in vacuo, treating the dipeptide as full QM (@ QM(VAC)), with the PB approach (@ PB(VAC)) and as full MM (@ MM(VAC)). The classical portions of the aminoacids are treated with the AMOEBA potential, while the QM systems are treated at the B3LYP/6-31G level of theory. The total energy is reported in millihartree (mE_H) and shifted by the average value computed along the trajectory ($\langle E_{tot}(t) \rangle_t$), while the time on the x axis is reported in pico-seconds (ps). ALA @ QM and ALA @ MM have been shifted by -0.1 mE_H and +0.1 mE_H respectively, to avoid the superposition of the different time series

S2 Extended Dynamics of the oligopeptide test systems

The total energy conservation along the extended dynamics of SAPPAS, in Fig. S2, and PEP, in Fig. S3, oligopeptides are reported.

In Fig. S2 the SAPPAS' energy time series for the dynamics in the water droplet for both the full MM and the QM/MM(PB) cases are shown, for a total simulation time of 6 ps. For the QM/MM(PB) dynamics the total energy stays close to that of the full MM one until ∼3 ps. After this time a linear negative drift starts to be noticeable. It is worth to note that the observed drift in the QM/MM(PB) dynamics at the end of the simulation amounts to ∼0.3 mE_H (less than 0.2 kcal/mol). The drift is not seen, or mach less remarkable, in the full MM trajectory.

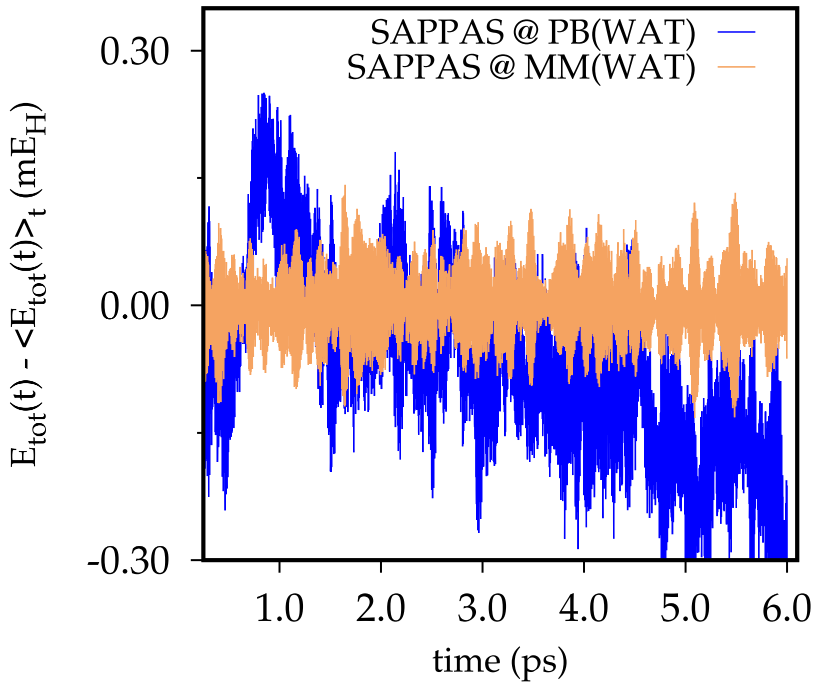

Figure S2: SAPPAS extended dynamics both in the QM/MM(PB) scheme (blue line) and in the full MM one (yellow)

In Fig. S3 are reported the energy time-series for the PEP QM/MM(PB) dynamics (blue), compared with the full MM one, both in the same droplet of AMOEBA water molecules. It

is mentioned here, as a reminder, that also the full MM oligopeptides are treated with the AMOEBA FF. A relatively larger energy fluctuation is observed during the 7.5 ps dynamics (in the order of 0.1 mE_H), if compared with those of the SAPPAS system, due to the larger dimension of the molecular system and the larger amount of solvent water molecules.

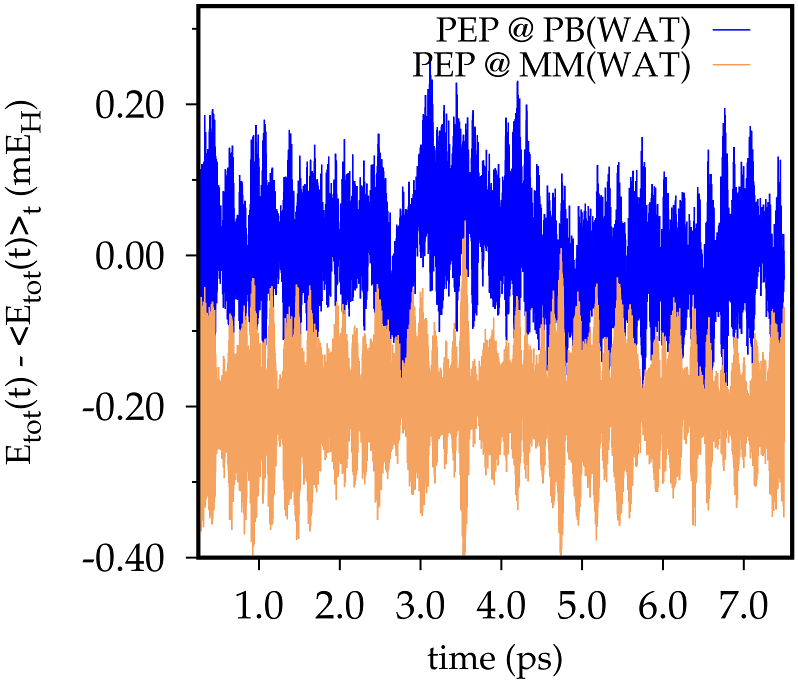

Figure S3: PEP extended dynamics, both in the QM/MM(PB) scheme (blue line) and in the full MM one (yellow)

S3 Excited State mixing on different structure with the B3LYP functional

The two QM/MM partition schemes described in the main text for the dynamics, namely the QM/MM and the QM/MM(PB) schemes, are here applied in a static approach to compute excitation properties. A set of snapshots are extracted from the MD_1 trajectory where the QM/MM(PB) scheme has been applied, and used as dye-environment configurations for the single point calculations at the TDB3LYP/6-31+G(d)/AMOEBA level. The scheme

for TDDFT simulation equivalent to the QM/MM(PB) one for dynamics is renamed here QM/MM-PB to avoid ambiguities between the dynamic scheme and the static one adopted for the excitation energy properties. This tests have been carried out to asses the combined effect of the new partition scheme and of the DFT functional on the excitation of interest, which is the $\pi\pi^*$ excitation localized on the TO chromophore.

Excitation energies are computed sampling the MD_1 trajectory every 80 fs, at the level of theory based on the B3LYP functional and consistent with that of Ref. 1.

The inclusion of the nucleobases (NBs) in the QM subsystem could introduce spurious excited states, other than that localized on the TO dye and responsible of its absorption and emission spectra. This can be seen in Fig. S4, where excitation energies (upper panel) and squared moduli of the electronic transition dipole moments (lower panel), computed on the MD_1 trajectory within the two partitioning schemes, are reported. Blue continuous lines show the $\pi\pi^*$ TO transition energy and dipole computed in the QM/MM scheme, while the dashed blue lines are their respective averages along the trajectory. The first three roots of the TDDFT equations for the QM/MM-PB scheme are analyzed, and in Fig. S4 the straight red line includes the excitations with the highest transition dipole moment per snapshot, and the closer energy to the corresponding excitation in the QM/MM scheme. The second and the third transitions found that exhibit a non negligible squared transition dipole moment (> 1 a.u., re) are also reported as red asterisks or squares. Such multiplication of states with week, but not negligible intensities, is present only in the QM/MM-PB partition. The presence of more than one state with non negligible intensity in the QM/MM-PB scheme needs to be investigated to select the right excitation for the present case. In Fig. S5 the natural transition orbitals (NTOs)[2] of the first three excitations computed with the QM/MM-PB scheme on one snapshot extracted from MD_1 are reported. A snapshot (number fifteenth in Fig. S4) showing one of the largest discrepancies in the transition dipole moments with respect to the QM/MM scheme has been chosen. In all the cases one nucleobase is involved in the transition, either contributing to the hole, as one guanine in S_1 and S_2, or to the

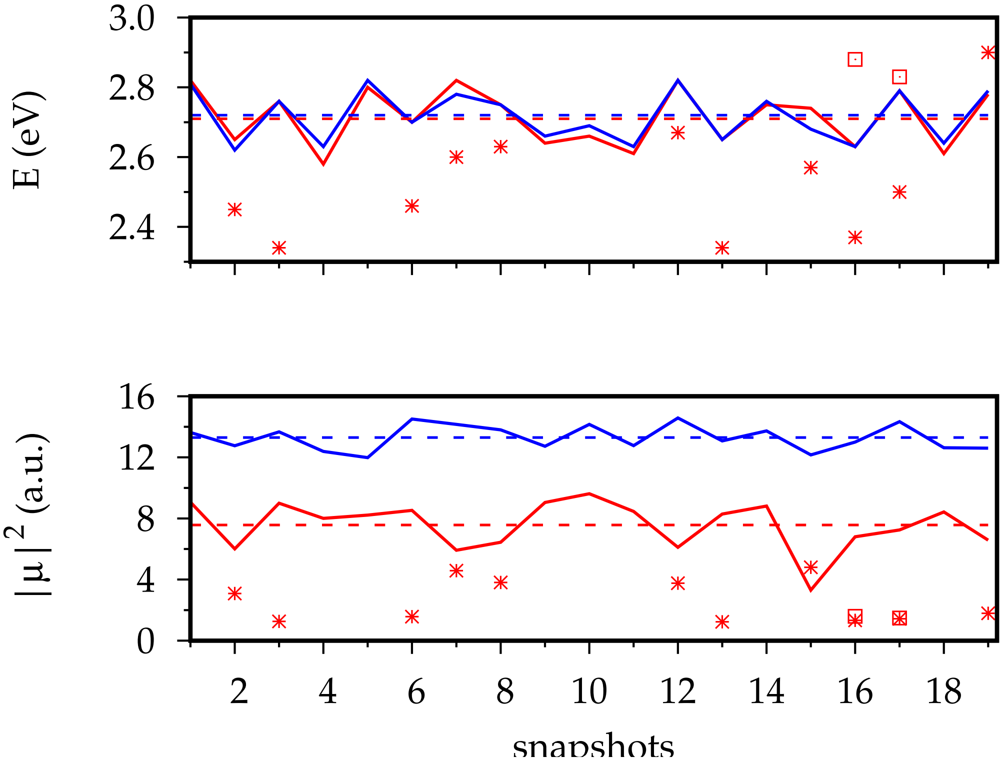

Figure S4: Comparison between QM/MM (blue lines) and QM/MM-PB (red lines) TDB3LYP/6-31+G(d)/AMOEBA results along the MD_1 QM/MM(PB) trajectory, where only the TO and its closest four nucleobases are QM and free to move during the dynamics. Excitation energies (upper panel) and transition dipole moments (lower panel) are reported. Continuous lines represent the bright transitions associated with the TO dye, while red squares and asterisks for the QM/MM-PB partition scheme represent energies and transition dipoles of the intruder excitations delocalized on the QM nucleobases. Dashed lines are the corresponding average values over the sampled snapshots

particle, as the cytosine in S_3. S_2 and S_3 transitions are closer in energy to the TO $\pi\pi^*$ found for the same snapshot in the QM/MM scheme. All the transitions analyzed are of $\pi\pi^*$ type, with a strong charge-transfer character. It has to be noticed that, in general, the electronic transition dipole moments computed with the QM/MM-PB scheme are smaller than those computed in the QM/MM one.

The QM/MM and QM/MM-PB partition schemes are both applied on two additional snapshots, extracted from one of the trajectories used to simulate the TO absorption spectrum in Ref. 1 and the results are reported in Tab. S1. The structures are extracted from a trajectory which shares the same starting point of MD_1, where the environment is kept frozen

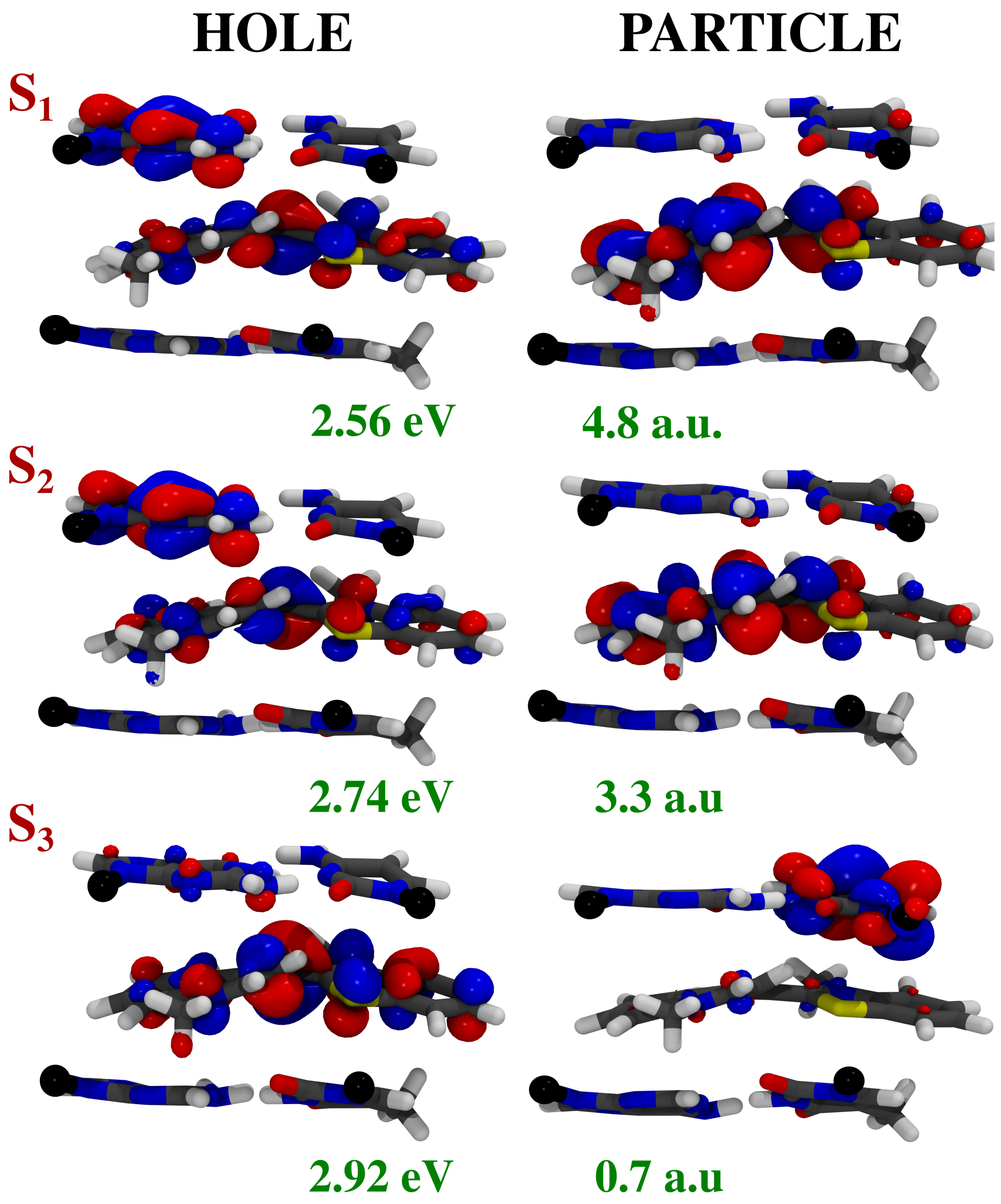

Figure S5: NTOs of the first three TDB3LYP roots in the QM/MM-PB partition scheme for excitation properties applied on one snapshot of the MD_1 QM/MM(PB) dynamics. The structure employed corresponds to one of the snapshots showing the largest disagreement between QM/MM and QM/MM-PB partitions for TDDFT calculations, whose compariso is reported in Fig. S4. S_1 is mainly composed by two pairs of MO (227 $\rightarrow$ 229, c = 0.286, and 228 $\rightarrow$ 229, c = 0.641) while three for S_2 (227 $\rightarrow$ 229, c = 0.641, 228 $\rightarrow$ 229, c = -0.272, and 228 $\rightarrow$ 230, c = -0.102) and one for S_3 (228 $\rightarrow$ 230, c = 0.690)

and the QM subsystem is composed by only the TO dye. It can be seen from the comparison that the mixing of electronic excited state in the QM/MM-PB is still observed. This shows that the state mixing is not an effect induced by the dynamics in the new QM/MM(PB) partition scheme, and particularly to the motion of the closest NBs, but more an effect related to the TDDFT approach and not strictly dependent on the geometry employed.

Table S1: Excitation energies and transition dipoles of the first three roots of the TDDFT equations computed on two snapshots extracted from the old trajectory reported in Ref. 1 and corresponding to MD_1. Two QM/MM partition schemes are reported, where the TO dye only is included in the QM subsystem (QM/MM), and where also the four closest nucleobases are treated at QM level (QM/MM-PB). The classical subsystem is always treated with the AMOEBA potential

	QM/MM-PB		QM/MM	
Snap 1	E (eV)	$\|\mu\|^2$ (a.u.)	E (eV)	$\|\mu\|^2$ (a.u.)
1	2.2	1.6	2.7	12.3
2	2.6	7.5		
3	3.2	0.1		
Snap 2	E (eV)	$\|\mu\|^2$ (a.u.)	E (eV)	$\|\mu\|^2$ (a.u.)
1	2.2	1.0	2.7	13.6
2	2.3	0.5		
3	2.7	8.4		

S4 DFT functional effects on the excitation properties

Excitation energies and oscillator strengths have been computed on the same snapshot extracted from the QM/MM(PB) MD_1 dynamics (see the manuscript for details), with different exchange-correlation functionals. From the pool of functionals chosen, mainly hybrid and range-separated functionals, it is possible to observe the effect of including long-range correction in the Coulomb interactions, improving the exciton quasiparticle (hole and particle pair) description.[3]

Table S2: Energies, in eV, of the first three roots of the TDDFT equations solved for the excited states of the TO embedded in DNA. In parenthesis also the oscillator strengths (in atomic units) are reported. The QM/MM and QM/MM-PB (see Section S3 for notation details) schemes for the excitation properties calculations are compared, starting from the same structures of MD_1. Also the difference between the excitation properties between the two methods, computed as the QM/MM-PB values minus the QM/MM ones (QM/MM-PB - QM/MM), are reported

State #	1	2	3
Functional	QM/MM-PB		
B3LYP	2.57 (0.3)	2.74 (0.2)	2.92 (0.0)
CAM-B3LYP	2.87 (0.6)	3.69 (0.0)	3.93 (0.0)
M062X	2.81 (0.6)	3.60 (0.0)	3.73 (0.0)
PBE0	2.57 (0.3)	2.74 (0.2)	2.92 (0.1)
ωB97X	2.97 (0.7)	4.18 (0.0)	4.32 (0.0)
ωB97XD	2.89 (0.6)	3.83 (0.0)	3.98 (0.0)
	QM/MM		
B3LYP	2.68 (0.8)	3.30 (0.0)	3.55 (0.0)
CAM-B3LYP	2.88 (0.9)	3.69 (0.0)	4.05 (0.0)
M062X	2.83 (0.9)	3.45 (0.0)	3.98 (0.0)
PBE0	2.74 (0.8)	3.50 (0.0)	3.68 (0.0)
ωB97X	2.98 (0.9)	4.19 (0.0)	4.37 (0.1)
ωB97XD	2.90 (0.9)	3.92 (0.0)	4.12 (0.1)
	QM/MM-PB - QM/MM		
B3LYP	-0.11 (-0.5)	-0.55 (0.2)	-0.63 (0.0)
CAM-B3LYP	-0.01 (-0.3)	0.00 (0.0)	-0.12 (0.0)
M062X	-0.02 (-0.3)	0.15 (0.0)	-0.25 (0.0)
PBE0	-0.17 (-0.5)	-0.76 (0.2)	-0.76 (0.0)
ωB97X	-0.01 (-0.3)	-0.01 (0.0)	-0.05 (-0.1)
ωB97XD	-0.01 (-0.3)	-0.09 (0.0)	-0.14 (0.0)

S5 Excitation properties computed sampling an additional configuration from the CMD

As mentioned in the manuscript (Section 4), a third set of trajectories have been performed, starting from a third dye-environment configuration extracted from the long classical molecular dynamics (CMD) of Ref 1. The results are reported in Table S3, compared with those

of the MD_2, which have been also reported in the main text. The comparison shows that the two set of trajectories give the same picture regarding the effect of the different QM/MM partitions and of the related dynamics on the excitation properties.

Table S3: Average values of the properties computed along the additional MD and MD_2 trajectories in the different QM/MM schemes are presented. Excitation energies (eV) and electronic transition dipole moments (in a.u.) are computed at TDCAM-B3LYP/6-31+G(d)/AMOEBA level. Each trajectory is sampled every 80 fs (as illustrated in the main text) to extract a set of system-environment configurations employed to compute the vertical excitations

	QM/MM		QM/MM(PB)	
	$<E_{exc}>$	$\|\mu\|^2$	$<E_{exc}>$	$\|\mu\|^2$
additional MD	2.88	14.1	2.83	10.7
MD_2	2.83	14.3	2.87	10.7
Diff.	0.05	-0.2	-0.04	0.04

S6 *mov*E excitations at different selection radii

In the main text is mentioned that, when the *mov*E scheme is applied, two different selection radii are tested. The selection radius is applied to reduce the number of explicit water molecules treated in the QM/MM MD and in the following TDDFT calculation. The selection radius is measured from each of the TO's QM atom, which is the center of a corresponding sphere. Each water molecule which falls inside the spheres defined in this way is kept in the classical description of the environment, treated with the AMOEBA potential and free to move during the dynamics of the QM system. In Table S4 the excitation energies for the two different radii explored are reported.

Table S4: Excitation energies for the two different radii used to select the moving classical polarizable environment in the *mov*E scheme. The excitation energies are reported in eV

	15 Å	23 Å
MD_1	2.810	2.818
MD_2	2.843	2.850

S7 Comparison of different QM subsystem sizes along the MD_1 trajectory

In the main text it is observed that there is generally a slight effect of an extended QM subsystem definition, going beyond the TO dye, on the excitation properties under investigation, if the structure of the whole system is kept the same and if the quality of the embedding used in computing the excitation is that offered by the AMOEBA FF. To confirm such statement, additional calculations are performed sampling the MD_1 trajectory, in the same way as it is done in the rest of this work, and the corresponding TDCAM-B3LYP/6-31+G(d) excitation energies are evaluated within the QM/MM and QM/MM-PB embedding schemes, following the notation of Section S3.

The results are reported in Fig. S6, showing that the differences between the QM/MM and the QM/MM-PB excitation, computed on the same structure of the TO-environment system, are always of the order of 10^{-2} eV, with the largest difference of 0.05 eV.

S8 AMOEBA Polarizable Vs Classical point-charges environment

In this section the explicit effect of the AMOEBA polarizable environment on the excitation properties evaluated at TDDFT level is compared with that of a classical, point-charges based FF. The amber99sb FF[4] for nucleic acids as implemented in the Tinker suite of programs has been chosen for the description of the DNA embedding, while the TIP3P[5] water model

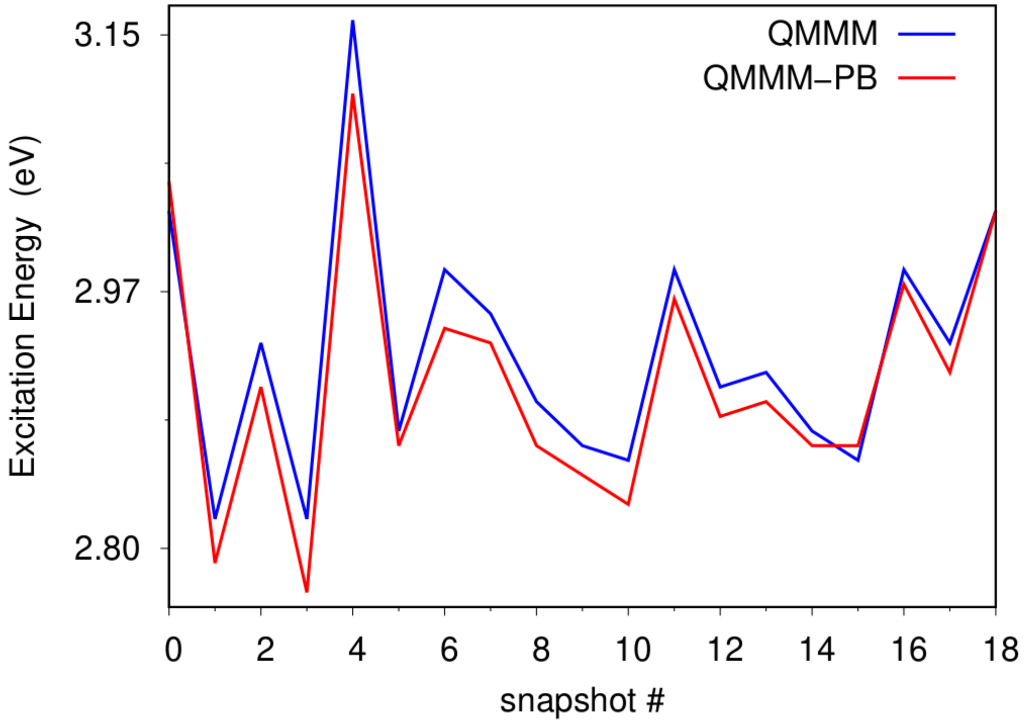

Figure S6

has been used for the solvent molecules.

The same trajectory (MD_1 QM/MM(PB)) has been sampled performing either QM/MM (only the TO dye is included in the QM subsystem) and QM/MM-PB (also the four NBs closest to the TO dye are treated at QM level) calculations, whose results are reported in Table S5. As it can be seen from the data reported in the Table, when the AMOEBA polarizable embedding is employed the difference in the average excitation energies computed within the two different QM/MM partitions are not significantly different (0.02 eV), while for the AMBER embedding their difference rise to a remarkable, even though not extremely high, 0.07 eV.

Table S5: Comparison between the TDCAM-B3LYP/6-31+G(d) excitation computed along the same QM/MM(PB) trajectory (MD_1 of the main text) using the non polarizable amber99sb embedding (AMBER) and AMOEBA. The excitations, toghether with their average value, are reported in eV

AMBER		AMOEBA	
QM/MM-PB	QM/MM	QM/MM-PB	QM/MM
3.08	3.13	3.05	3.03
2.84	2.91	2.79	2.82
2.97	3.07	2.91	2.94
2.82	2.91	2.77	2.82
3.07	3.19	3.11	3.16
2.92	3.00	2.87	2.88
2.99	3.07	2.95	2.99
2.99	3.06	2.94	2.96
2.93	2.99	2.87	2.90
2.90	2.98	2.85	2.87
2.86	2.93	2.83	2.86
3.03	3.13	2.97	2.99
2.94	2.99	2.89	2.91
2.97	3.06	2.90	2.92
2.93	3.00	2.87	2.88
2.92	2.96	2.87	2.86
3.03	3.11	2.98	2.99
2.94	2.97	2.92	2.94
3.07	3.13	3.03	3.03
Average			
2.96	3.03	2.91	2.93
Standard Deviation			
0.08	0.08	0.09	0.09

References

(1) Loco, D.; Jurinovich, S.; Cupellini, L.; Menger, M. F. S. J.; Mennucci, B. The modeling of the absorption lineshape for embedded molecules through a polarizable QM/MM approach. *Photochem. Photobiol. Sci.* **2018**, *17*, 552–560.

(2) Martin, R. L. Natural transition orbitals. *J. Chem. Phys.* **2003**, *118*, 4775–4777.

(3) Isborn, C. M.; Mar, B. D.; Curchod, B. F. E.; Tavernelli, I.; Martínez, T. J. The Charge Transfer Problem in Density Functional Theory Calculations of Aqueously Solvated Molecules. *J. Phys. Chem. B* **2013**, *117*, 12189–12201.

(4) Hornak, V.; Abel, R.; Okur, A.; Strockbine, B.; Roitberg, A.; Simmerling, C. Comparison of multiple Amber force fields and development of improved protein backbone parameters. *Proteins: Structure, Function, and Bioinformatics* **2006**, *65*, 712–725.

(5) Jorgensen, W. L.; Chandrasekhar, J.; Madura, J. D.; Impey, R. W.; Klein, M. L. Comparison of simple potential functions for simulating liquid water. *J. Chem. Phys.* **1983**, *79*, 926–935.
